# Characterization of protein marker expression, tumorigenicity, and doxorubicin chemoresistance in two new canine mammary tumor cell lines

**DOI:** 10.1186/s12917-014-0229-0

**Published:** 2014-09-30

**Authors:** Yen-Ling Hsiao, Tai-Zu Hsieh, Chian-Jiun Liou, Yeong-Hsiang Cheng, Chung-Tien Lin, Chi-Yao Chang, Yu-Shen Lai

**Affiliations:** Department of Biotechnology and Animal Science, National Ilan University, Yilan, Taiwan; Department and Graduate Institute of Veterinary Medicine, National Taiwan University, Taipei, Taiwan; Institute of Cellular and Organismic Biology, Academia Sinica, Taipei, Taiwan; Department of Nursing, Chang Gung University of Science and Technology, Taoyuan, Taiwan; Research Center for Industry of Human Ecology, Chang Gung University of Science and Technology, Taoyuan, Taiwan

**Keywords:** Canine mammary tumor, Cell line, Heat shock protein 27, Chemoresistance, Tumorigenicity

## Abstract

**Background:**

Canine mammary tumors (CMTs) are the most common type of cancer found in female dogs. Establishment and evaluation of tumor cell lines can facilitate investigations of the biological mechanisms of cancer. Different cell models are used to investigate genetic, epigenetic, and cellular pathways, cancer progression, and cancer therapeutics. Establishment of new cell models will greatly facilitate research in this field. In the present study, we established and characterized two new CMT cell lines derived from a single CMT.

**Results:**

We established two cell lines from a single malignant CMT specimen: DTK-E and DTK-SME. Morphologically, the DTK-E cells were large, flat, and epithelial-like, whereas DTK-SME cells were round and epithelial-like. Doubling times were 24 h for DTK-E and 18 h for DTK-SME. On western blots, both cell lines expressed cytokeratin AE1, vimentin, cytokeratin 7 (CK7), and heat shock protein 27 (HSP27). Moreover, investigation of chemoresistance revealed that DTK-SME was more resistant to doxorubicin-induced apoptosis than DTK-E was. After xenotransplantation, both DTK-E and DTK-SME tumors appeared within 14 days, but the average size of DTK-SME tumors was greater than that of DTK-E tumors after 56 days.

**Conclusion:**

We established two new cell lines from a single CMT, which exhibit significant diversity in cell morphology, protein marker expression, tumorigenicity, and chemoresistance. The results of this study revealed that the DTK-SME cell line was more resistant to doxorubicin-induced apoptosis and exhibited higher tumorigenicity *in vivo* than the DTK-E cell line. We anticipate that the two novel CMT cell lines established in this study will be useful for investigating the tumorigenesis of mammary carcinomas and for screening anticancer drugs.

## Background

Mammary gland carcinomas are the most common neoplasms in both women and female dogs [1,2]. Clinical evidence has shown that approximately 50% of canine mammary tumors (CMTs) are diagnosed as malignant in dogs [3]. CMTs possess epidemiological, clinicopathological, and biochemical characteristics similar to those of human breast carcinomas [4,5]. Therefore, CMTs have been considered an appropriate and valid model for human breast cancer studies for more than three decades [6].

Tumor cell lines have been widely used for *in vitro* research, and have proven to be a particularly useful tool for genetic analyses. Previous characterizations of tumor cell lines have shown that they are also excellent models for studying the biological mechanisms underlying cancer [7]. The use of tumor cell lines has increased our knowledge of the deregulated genes and signaling pathways involved in cancer [8,9]. Furthermore, original cell models have been developed to test anticancer drugs [8,10–12]. Tumor cell lines continue to be used in the development of new therapies [7,11,13], and also provide an alternative to direct transplantation of tumors in animals for testing chemotherapeutics [14].

The use of an appropriate *in vitro* model is crucial in cancer research. Different cell models are used to investigate genetic, epigenetic, and cellular pathways [7]; proliferation deregulation, apoptosis, and cancer progression [9]; and identification of potential molecular markers [15], as well as to screen and characterize cancer therapeutics [11,16]. The findings reported from tumor cell lines are typically extrapolated to *in vivo* human tumors [15]. Thus, many biomedical and pharmaceutical companies have recognized the importance of tumor cell lines as models for drug testing and translational studies [8].

In the present study, we established and characterized two new CMT cell lines, termed DTK-E and DTK-SME. These two cell lines exhibited significant diversity in cell morphology, protein marker expression, tumorigenicity, and chemoresistance. Our results suggest that the two established cell lines might constitute a useful experimental model for investigating the tumorigenesis of mammary carcinomas and for screening potential anticancer drugs.

## Results

### Morphological analysis of established cell lines

**Figure 1:**
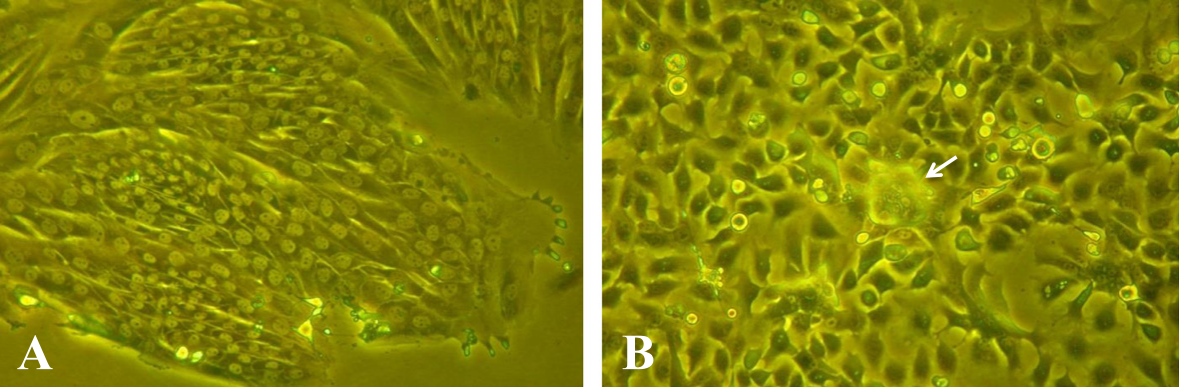
**Phase contrast micrographs of CMT cells derived from malignant tumor tissue. (A)** DTK-E cells displayed mostly large, flat, epithelial-like characteristics; **(B)** DTK-SME cells displayed rounded, epithelial-like features and possessed pile up. Arrowhead indicates position of pile up.

**Figure 2:**
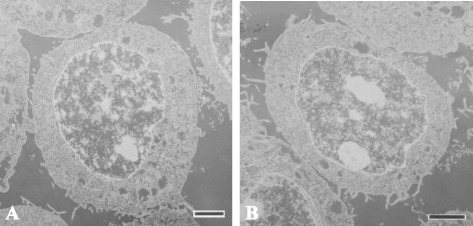
**Transmission electron micrographs of CMT cell lines.** Intracellular morphology of **(A)** DTK-E and **(B)** DTK-SME cells. Images are representative of 20 random sections per cell line. Scale bars = 2 μm.

After over 100 passages, two CMT cell lines, DTK-E and DTK-SME, were established from canine mammary carcinomas. When adhered to a culture plate, DTK-E cells exhibited a large, flat, epithelial-like morphology (Figure 1A), while DTK-SME cells presented a rounded, epithelial-like morphology (Figure 1B). Of the two established cell lines, only DTK-SME cells possessed pile up (Figure 1B). Transmission electron micrographs showed that both DTK-E and DTK-SME cells exhibited high nuclear-cytoplasmic ratios and large nucleoli (Figure 2).

### Biological analysis of established cell lines

**Figure 3:**
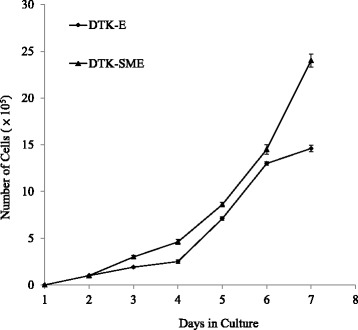
**Growth curves of DTK-E and DTK-SME cell lines.** Symbols represent the mean of triplicate samples and the data are expressed as the mean ± SE.

Alterations in the biological properties of the two CMT cell lines were monitored during their establishment. During the first 10 passages, 50% fetal bovine serum (FBS) was required to maintain cell growth. After 10 passages, the percentage of FBS in the medium was gradually reduced. After 100 passages, 5% FBS was sufficient to support the growth of both CMT cell lines. In addition, contact inhibition was only significant during the first 35 passages, and was completely lost after 50 passages (data not shown). During establishment of the two CMT cell lines, we also determined the doubling time. The final doubling times for DTK-E and DTK-SME cells were 24 h and 18 h, respectively (Figure 3).

### Expression profile of CMT genes and proteins

**Figure 4:**
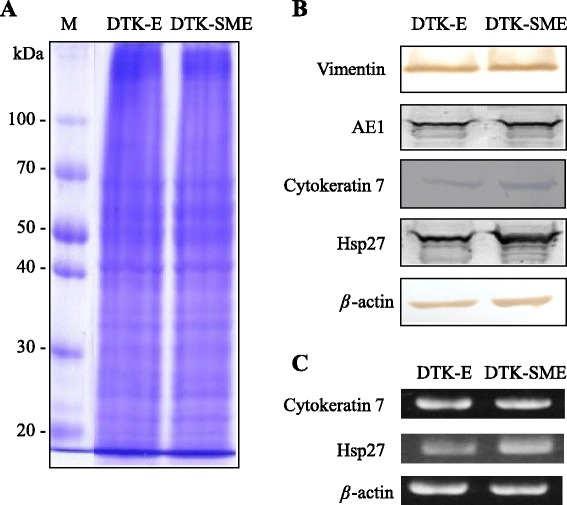
**Expression profiles of protein markers in the DTK-E and DTK-SME cell lines. (A)** Western blot analysis of total proteins in cell lysates. Lane 1, pre-stained protein marker; Lane 2, DTK-E; Lane 3, DTK-SME. **(B)** Protein expression in DTK-E and DTK-SME cell lines detected with antibodies to vimentin, cytokeratin 7 (CK7), low-molecular weight cytokeratin AE1, and heat shock protein 27 (HSP27). **(C)** RT-PCR analysis of *CK7* and *HSP27* gene expression in DTK-E and DTK-SME cell lines. *β-actin* served as an internal control.

We used western blot analysis with specific antibodies to investigate the individual protein expression profiles of the two CMT cell lines (Table 1). Both CMT cell lines expressed the low-molecular weight cytokeratin AE1 and vimentin, cytokeratin 7 (CK7), and heat shock protein 27 (HSP27) (Figure 4A, B). In addition, DTK-SME exhibited a higher level of HSP27 expression than DTK-E cells. In reverse transcription-polymerase chain reaction (RT-PCR) assays, the *CK7* and *HSP27* gene expression profiles were the same as their respective protein expression profiles; both DTK-E and DTK-SME cells expressed *CK7* and *HSP27* genes (Figure 4C).

**Table 1:** **Overview of the sources and dilutions of the primary antibodies used in this study**

**Antibody**	**Source**	**Clone**	**Dilution**
AE1	NeoMarkers	AE1 (mouse monoclonal)	1:1000
Vimentin	NeoMarkers	V-9 (mouse monoclonal)	1:1000
CK 7	Santa Cruz	5F282 (mouse monoclonal)	1:1000
Hsp27	GeneTex	GTX25579 (rabbit polyclonal)	1:1000
Caspase 3	NeoMarkers	CPP (rabbit polyclonal)	1:1000
β-actin	Sigma	AC-15 (rabbit polyclonal)	1:5000

### Tumorigenicity of established CMT cell lines

**Figure 5:**
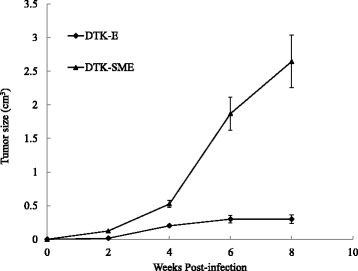
**Tumor growth curves of DTK-E and DTK-SME cells in tumorigenicity tests.** Cells were subcutaneously injected into nude mice and monitored for 8 weeks. The tumor volumes were estimated every 2 weeks and calculated, and the data are expressed as the mean ± SE.

We performed xenografts to examine the tumorigenicity of the two CMT cell lines. At passage 100, each cell line was injected subcutaneously into five 8-week-old athymic mice (BALB/c *nu/nu*). A tumor mass was first observed at the injection site in all mice injected with DTK-E and DTK-SME cells at 1 week post-injection; at 8 weeks, the DTK-SME and DTK-E groups exhibited masses of 1.8–2.2 cm and 0.5–0.7 cm in diameter, respectively (Figure 5).

### Doxorubicin (DOX)-induced dose-dependent growth suppression, apoptosis, and caspase-3 activation in CMT cells

**Figure 6:**
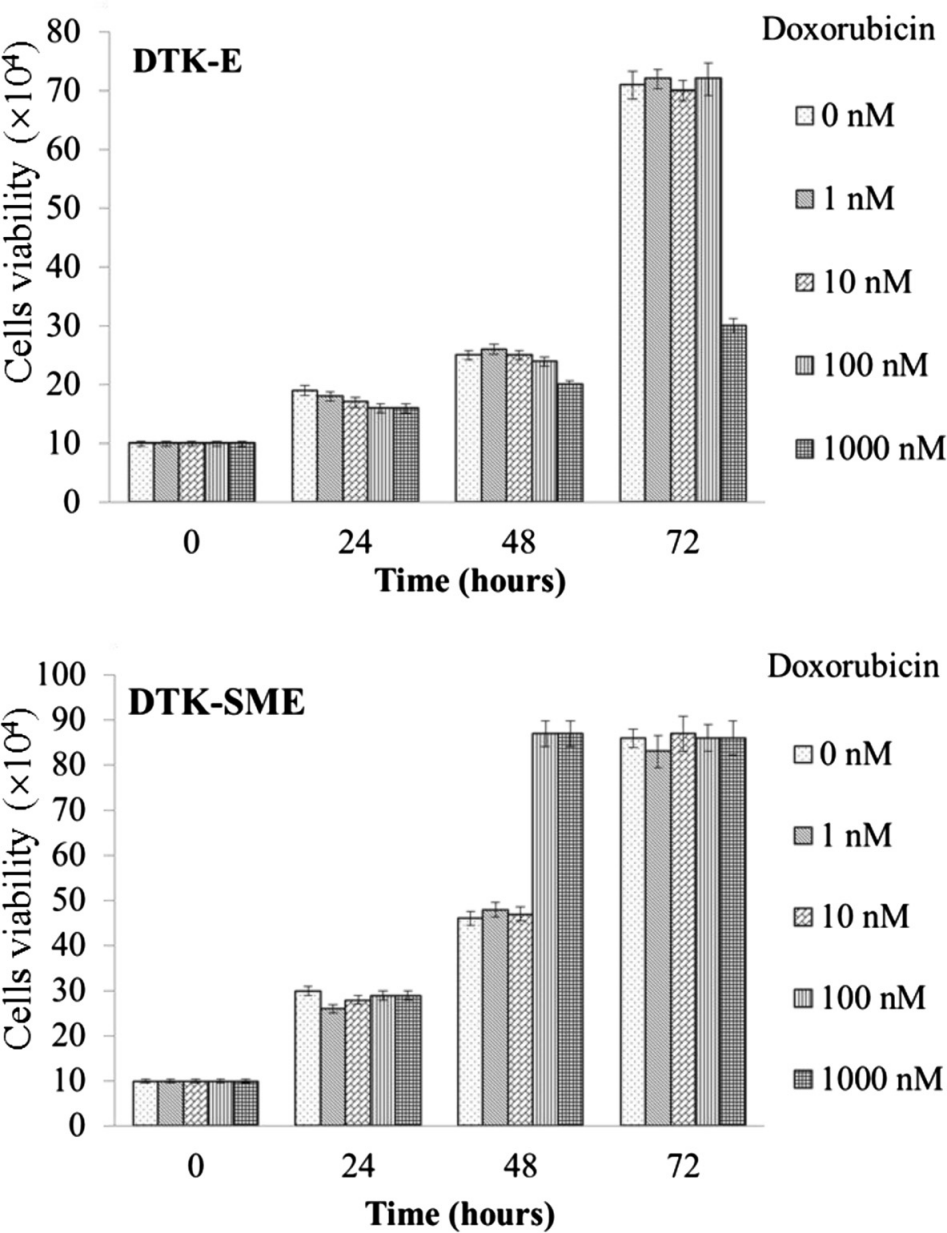
**Effects of DOX on DTK-E and DTK-SME cell proliferation.** Cells were treated with escalating doses of DOX (1, 10, 100, and 1000 nM) and incubated for 24 h, 48 h, and 72 h. Cell viability was determined every 24 h. Cell numbers were averaged over triplicate samples, and the data are expressed as the mean ± SE.

**Figure 7:**
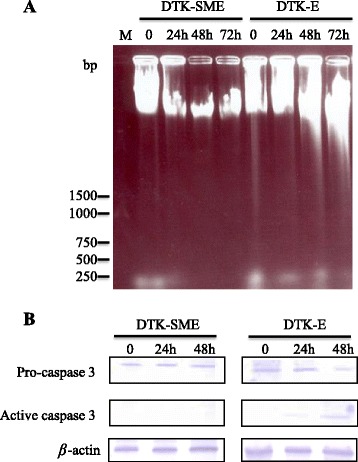
**DOX-induced apoptosis and caspase-3 activation in DTK-E and DTK-SME cells. (A)** Agarose gel electrophoresis of total cellular DNA showed DOX-induced DNA fragmentation (apoptosis) in CMT cells. Cells were incubated with 1000 mM DOX for 24 h, 48 h, and 72 h. M: DNA marker. **(B)** Western blots show DOX-induced activation of caspase-3 in CMT cells. Cells were incubated with 1000 nM DOX for 24 h and 48 h.

Next, we investigated the susceptibility of the two CMT cell lines to DOX. Cells were treated with DOX and cell growth and apoptosis were analyzed at 24 h, 48 h, and 72 h. We found that DOX (1000 nM) was able to promote apoptosis and inhibit proliferation in a dose- and time-dependent manner in DTK-E cells, but not in DTK-SME cells (Figures 6 and 7). Further quantitative analysis revealed that 72-h exposures to 100 nM and 1000 nM DOX resulted in 2% and 58% mortality, respectively, in DTK-E cells (Figure 6). In addition, DOX (1000 nM) treatment caused DNA fragmentation in DTK-E cells at 72 h (Figure 7A). In contrast, no DNA fragmentation was observed at 72 h in DTK-SME cells. Because activation of caspase-3 is a hallmark of apoptosis, we examined caspase-3 activation in DOX-treated (1000 nM) CMT cells (Figure 7B). DOX induced the time-dependent cleavage of procaspase-3 into its active caspase-3 form in DTK-E cells, but not in DTK-SME cells.

## Discussion

In the present study, we established and characterized two new CMT cell lines derived from a single malignant tumor: DTK-E and DTK-SME. DTK-E cells showed a large, flat, epithelial-like morphology, while DTK-SME cells showed a rounded epithelial-like morphology. Both DTK-E and DTK-SME cells exhibited a high nuclear-cytoplasmic ratio, which is often associated with malignancy in tumor cells. Consistent with this finding, xenografts of injected DTK-E and DTK-SME cells developed into prominent solid tumors within 2 weeks, and the tumor mass of DTK-SME was larger than that of DTK-E after the 8-week study.

The human breast contains a branching ductal network composed of two epithelial cell types: an inner layer of polarized luminal epithelial cells and an outer layer of myoepithelial cells. Breast cancer arises mainly in the luminal epithelial compartment of the terminal duct lobular units [17,18]. Several proteins have been identified as important cell markers for investigating tumor pathophysiology. Cytokeratins (CKs) are critical markers of epithelial differentiation [19]. The distribution of CKs is tissue-specific, and this feature facilitates the determination of tumor origin [20–22]. One low-molecular weight cytokeratin subtype, CK7, is a marker of luminal epithelial cells in both human and canine mammary tissues [23]. In a study of 435 cases, expression of CK7 was associated with up to 96% of human breast epithelial neoplasms [24]. Other studies have also reported a high incidence of CK7 (56%) in canine mammary gland carcinomas [25]. In this study, we showed that DTK-E and DTK-SME expressed CK7 and possessed high tumorigenicity. These results suggest that the DTK-E and DTK-SME cell lines could have originated directly from the tumor cells in the surgical specimen.

Vimentin is an intermediate filament protein expressed in mesenchymal cells [26]. Vimentin expression in invasive breast carcinomas is generally considered to indicate the epithelial-to-mesenchymal transition or myoepithelial histogenesis. Vimentin-positive breast tumors have been described as mostly malignant, highly invasive, and chemoresistant [27]. Interestingly, the DTK-E and DTK-SME cell lines established in this study were vimentin-positive and displayed tumorigenicity in mice.

HSP27 is a member of the small heat shock protein family of molecular chaperones. It plays an essential role in protein folding and protects cells from stress-induced damage [28,29]. In general, the healthy canine (and human) mammary gland exhibits little or no HSP27 expression [30–32]. In human breast cancer tumor cells, overexpression of HSP27 was associated with accelerated cancer progression [33], increased anchorage-independent growth [34], increased invasiveness [35,36], and resistance to chemotherapeutic drugs [34,37–39]. In patients, elevated expression of HSP27 in human breast cancer tissues has been shown to indicate poor prognosis and a low survival rate [40,41]. Therefore, increased expression of HSP27 in human breast cancers might affect the prognosis and treatment outcome [42].

DOX is an antitumor drug that can promote tumor cell apoptosis. Currently, DOX is widely used to treat a broad spectrum of cancers. *In vitro* studies of cancer cells have shown that HSP27 expression protects against apoptosis and confers resistance to DOX [38,43,44]. Interestingly, we also observed an association between increased HSP27 expression and resistance to DOX (Figures 6 and 7). Indeed, HSP27 expression was elevated in both the DTK-E and DTK-SME cell lines, although the expression level was higher in DTK-SME cells. Accordingly, DTK-SME cells were more resistant to DOX-mediated apoptosis and tumor masses were larger than those of DTK-E cells. The correlation between HSP27 and CMT malignancy currently remains unclear; thus, these two CMT cell lines with different tumorigenicities may serve as a valuable cell model for future studies of the roles of HSP27 in tumorigenicity and chemoresistance.

## Conclusion

We established and characterized two new CMT cell lines with diverse tumorigenic properties. DTK-E cells were large, flat, and epithelial-like, while DTK-SME cells showed a rounded epithelial-like morphology and possessed pile up. Both DTK-E and DTK-SME cells were vimentin^+^/AE1^+^/CK7^+^/HSP27^+^ and tumorigenic in nude mice. These two cell lines also exhibited differential susceptibility to DOX-mediated apoptosis. Based on their diverse malignancy, tumorigenicity, protein marker profiles, and drug susceptibility, these newly established CMT cell lines represent useful cell models for basic tumor biology studies and for developing antitumor drugs to treat both human and canine breast carcinomas.

## Methods

### Tumor specimen

The tumor tissues used in this study were obtained from client-owned dogs with the consents of the owners acquired prior to sample collection. The handling of animals was practiced following a high standard of veterinary care. A tumor specimen was surgically excised from the mammary tissue of an 8-year-old mixed-breed female dog. Fresh neoplastic masses from the specimen were placed in primary culture, and the remaining samples were fixed in 10% formalin for histopathology. A histological examination confirmed the diagnosis of mammary carcinoma. Tumor specimens consisted of highly proliferating, luminal epithelial and fibroblastic stromal cells; they also had glandular structures with irregular lumens embedded in fibrous stroma.

### Establishment of cell lines

A single tumor specimen was washed with RPMI 1640 medium (Sigma; St. Louis, MO, USA) that contained 500 IU/mL penicillin and streptomycin. The tissue was minced with scissors and transferred to a 60-mm-diameter tissue culture dish (Nunc; Waltham, MA USA) containing 10 mL of 0.25% trypsin solution (0.25% trypsin and 0.2% ethylenediaminetetraacetic acid [EDTA] in phosphate-buffered saline [PBS]). The tissue suspension was gently agitated with a magnetic stir bar at 4°C for 1 h. The resulting supernatant was transferred to a new tube. An equal volume of complete RPMI 1640 medium, containing 10% FBS (Hyclone Laboratories; Logan, UT, USA) and 100 IU/mL penicillin and streptomycin was added and mixed well. The mixture was centrifuged at 180 × *g* for 5 min, and the pelleted cells were resuspended in fresh, complete medium. The cells were then seeded into 25-cm^2^ culture flasks and incubated with complete medium at 37°C in a humidified atmosphere of 95% air and 5% CO_2_. Observations were performed daily with a phase-contrast microscope. When cultured cells reached 90% confluence, they were washed with 2 mM EDTA in PBS, dispersed in the 0.25% w/v trypsin solution, and placed in a new flask at a density of 1 × 10^5^ cells/mL. After 20 passages, cells were stored in RPMI 1640 medium containing 20% FBS and 10% dimethyl sulfoxide (Sigma). In addition, at passage 20, different cell types were subcloned from the parental cell line with a limiting-dilution method.

### Electron microscopy

After 100 passages, cultured CMT cells were harvested, fixed with 2.5% glutaraldehyde in 0.1 M cacodylate buffer, and placed at 4°C overnight. After fixation, the cells were treated with 1% osmium tetroxide at 4°C for 2 h, washed in cacodylate buffer, dehydrated in graded acetone solutions, and embedded in Spur’s low-viscosity resin. The embedded specimen was sectioned with a Reichert-Jung Ultracut E ultramicrotome (Nussloch, Germany), and the sections were stained with 2% uranyl acetate (EMS; Fort Washington, PA, USA) in distilled water for 1 min. Images were acquired with a JEOL JEM 2000 EXII transmission electron microscope (JEOL Ltd.; Tokyo, Japan).

### Growth assay

To investigate the growth of CMT cells, after 100 passages, 1 × 10^5^ cells were seeded in 25-cm^2^ tissue culture flasks and incubated in complete medium at 37°C. After the cells were detached with trypsinization, they were stained with trypan blue in triplicate flasks and counted with a hemocytometer. The average number of cells was calculated and recorded.

### Western blot analysis

CMT cells were collected and lysed with 100 μL sample buffer that contained 100 mM Tris–HCl buffer, pH 6.8, 4% sodium dodecyl sulfate (SDS), 0.07% β-mercaptoethanol, 20% glycerol, and 0.2% bromophenol blue. The crude cell lysate proteins were electrophoresed on a 12% SDS-acrylamide gel and then stained with Coomassie Brilliant Blue R-250 for protein profiling or transferred to polyvinylidene fluoride membranes for western blot analysis. The membranes were blocked with 5% skim milk, incubated with primary antibodies (Table 1) for 2 h, and subsequently incubated with alkaline phosphatase-conjugated secondary antibodies. Membranes were developed with 5-bromo-4-chloro-3-indolyl phosphate and 4-nitro-blue tetrazolium chloride as substrates.

### Total RNA isolation and RT-PCR

Total RNA was isolated from the cells using TRIzol reagent (Invitrogen; Paisley, Scotland, UK) according to the manufacturer’s protocol. After digestion with RNase-free DNase (New England Biolabs; Beverly, MA, USA), 2 μg of total RNA was reverse-transcribed into first-strand cDNA using random primers according to the Reverse Transcriptase Kit (Roche; Penzberg, Germany) protocol. The PCR was carried out in a volume of 50 μL containing 2 μL cDNA, 0.5 μM forward primer, 0.5 μM reverse primer, 2.5 μM dNTP, 1× PCR buffer, and 2.5 U Taq DNA polymerase (Viogene; Taipei, Taiwan). The primers for *CK7* were as follows: Forward-5′- CAGGTGCGCCTGAGCTCG-3′, Reverse-5′-GCGGTAGGTGGCGATCTC-3′. PCR was carried out under the following conditions: 10 min at 95°C; 35 cycles of 1 min at 95°C, 1 min at 56°C, and 1 min at 72°C; and 10 min at 72°C. The primers for *HSP27* were as follows: Forward-5′-ATGACCGAGCGCCGAGTGCCC-3′, Reverse-5′-CTTGGCTCCAGACTGCTCCGA-3′. PCR was carried out under the following conditions: 5 min at 95°C; 35 cycles of 45 s at 95°C, 45 s at 54°C, and 1 min at 72°C; and 10 min at 72°C. The primers for *β-actin* were as follows: Forward-5′-CTGGGACGACATGGAGAA-3′, Reverse-5′-GAGTACTTGCGCTCAGG-3′. PCR was carried out under the following conditions: 5 min at 95°C; 35 cycles of 45 s at 95°C, 45 s at 55°C, and 45 s at 72°C; and 10 min at 72°C. The PCR products were electrophoresed on a 1.2% agarose TAE-buffered gel and stained with ethidium bromide.

### Tumorigenicity

Animal handling and experimental procedures were approved by the Institutional Animal Care and Use Committee of National Ilan University (Reference number: No. 103-10). After 100 passages, CMT cells (1 × 10^7^) were subcutaneously inoculated into the backs of 8-week-old athymic BALB/c nude mice. Five nude mice were used to test each cell line. Physiological data of the mice were recorded weekly, and tumor volumes were estimated every 14 days and calculated as described previously [45]. Mice were sacrificed 56 days after inoculation. Results are expressed as the mean tumor volume.

### Susceptibility to doxorubicin

After 100 passages, CMT cells (1 × 10^5^) were seeded into 25-cm^2^ tissue culture flasks in triplicate. After 24-h incubation, cells were treated with the antitumor drug DOX for 72 h. Mock control cells were treated with water. After the treatment, cells were trypsinized, harvested, stained with 0.4% trypan blue, and counted with a hemocytometer under a light microscope. The average number of cells in triplicate flasks was calculated and recorded.

### DNA ladder assay

After DOX (1000 nM) treatment, cells were collected and lysed at 55°C for 1 h in 500 μL lysis buffer that contained 100 mM NaCl, 10 mM Tris (pH 8.0), 25 mM EDTA, 0.5% SDS, 200 μg/mL DNase-free proteinase K (Sigma), and 50 μg/mL DNase-free RNase (Sigma). DNA was extracted with phenol/chloroform, precipitated with 100% ethanol, and resuspended in double-distilled water. DNA samples were then separated on l.6% agarose gels and stained with ethidium bromide.

## Abbreviations

CMT: Canine mammary tumor
CK: Cytokeratin
CK7: Cytokeratin 7
HSP27: Heat shock protein 27
DOX: Doxorubicin
FBS: Fetal bovine serum
EDTA: Ethylenediaminetetraacetic acid
PBS: Phosphate-buffered saline
SDS: Sodium dodecyl sulfate
